# Soluble CD59 Expressed from an Adenovirus *In Vivo* Is a Potent Inhibitor of Complement Deposition on Murine Liver Vascular Endothelium

**DOI:** 10.1371/journal.pone.0021621

**Published:** 2011-06-24

**Authors:** Jarel Gandhi, Siobhan M. Cashman, Rajendra Kumar-Singh

**Affiliations:** Department of Ophthalmology, Tufts University School of Medicine, Boston, Massachusetts, United States of America; Katholieke Universiteit Leuven, Belgium

## Abstract

Inappropriate activation of complement on the vascular endothelium of specific organs, or systemically, underlies the etiology of a number of diseases. These disorders include atypical hemolytic uremic syndrome, membranoproliferative glomerulonephritis, atherosclerosis, age-related macular degeneration, diabetic retinopathy, and transplant rejection. Inhibition of the terminal step of complement activation, i.e. formation of the membrane attack complex, using CD59 has the advantage of retaining the upstream processes of the complement cascade necessary for fighting pathogens and retaining complement's crucial role in tissue homeostasis. Previous studies have shown the necessity of membrane targeting of soluble CD59 in order for it to prove an effective inhibitor of complement deposition both *in vitro* and *in vivo*. In this study we have generated an *in vivo* model of human complement activation on murine liver vascular endothelium. This model should prove useful for the development of anti-complement therapies for complement-induced pathologies of vascular endothelium. Using this model, we have demonstrated the viability of a *non* membrane-targeted soluble CD59 to significantly inhibit complement deposition on the endothelium of murine liver vasculature when expressed *in vivo* from an adenovirus. This result, unanticipated based on prior studies, suggests that the use of non membrane-targeted sCD59 as an anti-complement therapy be re-visited.

## Introduction

Complement is a key component of innate immunity [1]. Activation of complement results in the generation of anaphylatoxins - pleiotropic effector molecules that mediate both inflammatory processes, such as chemoattraction, vasodilation and vasopermeability. In addition, anaphylatoxins mediate non-inflammatory processes, such as tissue regeneration, lipid metabolism, and synapse formation (reviewed in [2]). Activation of complement terminates in the formation of a pore on the surface of target cells referred to as the membrane attack complex (MAC), resulting in cell lysis.

Inappropriate activity of the complement system, specifically on endothelial cells, results in a number of diseases. For example, damage and detachment of the endothelium due to abnormal complement activity has been documented in atypical hemolytic uremic syndrome (aHUS) [3]. Both types of membranoproliferative glomerulonephritis (MPGN), type I and dense deposit disease (MPGN type II), are characterized by the presence of complement proteins within the subendothelial dense-deposit along the glomerular basement membrane [4]. Dense deposit disease has been linked to a deficiency in the complement regulator, Factor H [5]. Transgenic mice expressing a negative regulator of complement, referred to as protectin (CD59), on the endothelium are protected against atherosclerosis [6]. Age-related macular degeneration (AMD) has been tightly linked to polymorphisms in various complement genes [7], and complement proteins such as MAC have been observed to be deposited on choroidal endothelial cells [8]. Complement proteins have also been documented to be deposited on the choriocapillaris of patients with diabetic retinopathy, as well as in the retinal vessels of diabetic subjects [9]. These vessels also exhibited a significant reduction in expression of the complement regulatory proteins, decay accelerating factor (CD55) and CD59. Hyperacute rejection of organ transplantation, mainly the liver and kidney, has shown evidence of complement activity on the endothelium, and is considered a key reason for transplant rejection [10]. An *ex vivo* perfusion simulation of xenotransplantation using normal human blood in porcine liver has indicated intralobular hemorrhage and complete loss of hepatic function within hours of complement component 3 and MAC deposition on endothelial cells [11]. Ischemia/reperfusion (I/R) injury, a complication of a number of pathologies including organ transplantation, stroke, myocardial infarction, sepsis and shock, has been shown to be mediated by complement-induced damage of endothelial cells of the vasculature and a number of studies have shown the effectiveness of complement inhibitors to reduce I/R injury (reviewed in [12]).

CD59 is a glycosylphosphatidyl inositol (GPI)-anchored membrane inhibitor of MAC formation on cellular membranes [13], [14]. Studies have shown that while the activity of a soluble version of the inhibitor of MAC formation (sCD59) is one hundred times lower than that of GPI-linked CD59 in the absence of human serum *in vitro* [15], in the presence of serum concentrations as high as 50% the activity of sCD59 was 10-fold higher than that of GPI-linked CD59. These studies suggest sCD59 as a viable alternative to the membrane-bound form of CD59 *in vivo*. Multiple groups, however, have reported that recombinant sCD59 alone is a poor therapeutic molecule, as they have not observed any significant protection from complement-mediated damage to cells or tissues either in *in vitro* or *in vivo*. To overcome this poor performance, sCD59 chimeras that target recombinant sCD59 to the cell membrane have been developed. A soluble form of rat CD59 fused with a plasma membrane-addressing peptide (APT542) has been shown to reduce MAC activity *in vivo* in a rat model of rheumatoid arthritis [16]. When rat sCD59 is delivered as a recombinant Fc fusion protein, protection against MAC-induced damage has been successful in both a model of antigen-induced arthritis and of laser-induced choroidal neovascularization [17], [18]. Soluble CD59 fused with either a fragment of complement receptor 2 which targets the protein to activated complement proteins deposited on cell membranes, or to an IgG, provided protection from MAC on chinese hampster ovary cells *in vitro* [19], [20].

In contrast to the above *in vivo* studies which involved delivery of a recombinant membrane-targeting form of sCD59, we have recently shown that a membrane-*in*dependent sCD59 when expressed *in vivo* in murine ocular tissue via an adenovirus or adeno-associated virus (AAV) vector can significantly reduce MAC deposition and laser-induced choroidal neovascularization in a mouse model of neovascular AMD [21]. We postulated that the success of that study may be explained in part by constitutive expression of high concentrations of sCD59 in the closed ocular environment. To determine whether this gene therapy approach was also applicable to other tissues, we sought to examine whether we could inhibit MAC deposition on endothelial cells of murine liver vasculature *in vivo* using sCD59. To address this, we generated an *in vivo* model of human complement deposition on liver vascular endothelium. We employed an approach similar to that previously described by us in which we generated an *ex vivo* model of human MAC deposition on murine retinal pigment epithelial cells (RPE) [22], a model which has proven useful for validating the potential of human membrane-linked complement regulators (CD59 [22], CD55 [23], and CD46 [24]) to protect RPE cells against attack by human complement. By infusion of an endothelial cell-specific antibody followed by human serum, we have been able to achieve human MAC deposition on the endothelium of murine liver vasculature. This model should prove useful in the rapid testing of anti-complement therapies for the treatment of complement-mediated diseases of the vasculature. We then tested and confirmed the capacity of an adenovirus-delivered sCD59 to protect liver vascular endothelium from human MAC deposition. Our study suggests that the use of a membrane-*in*dependent sCD59 as an anti-complement therapy for complement-associated diseases should be re-visited.

## Results

### Deposition of human membrane attack complex on murine endothelial cells *ex vivo*

**Figure 1:**
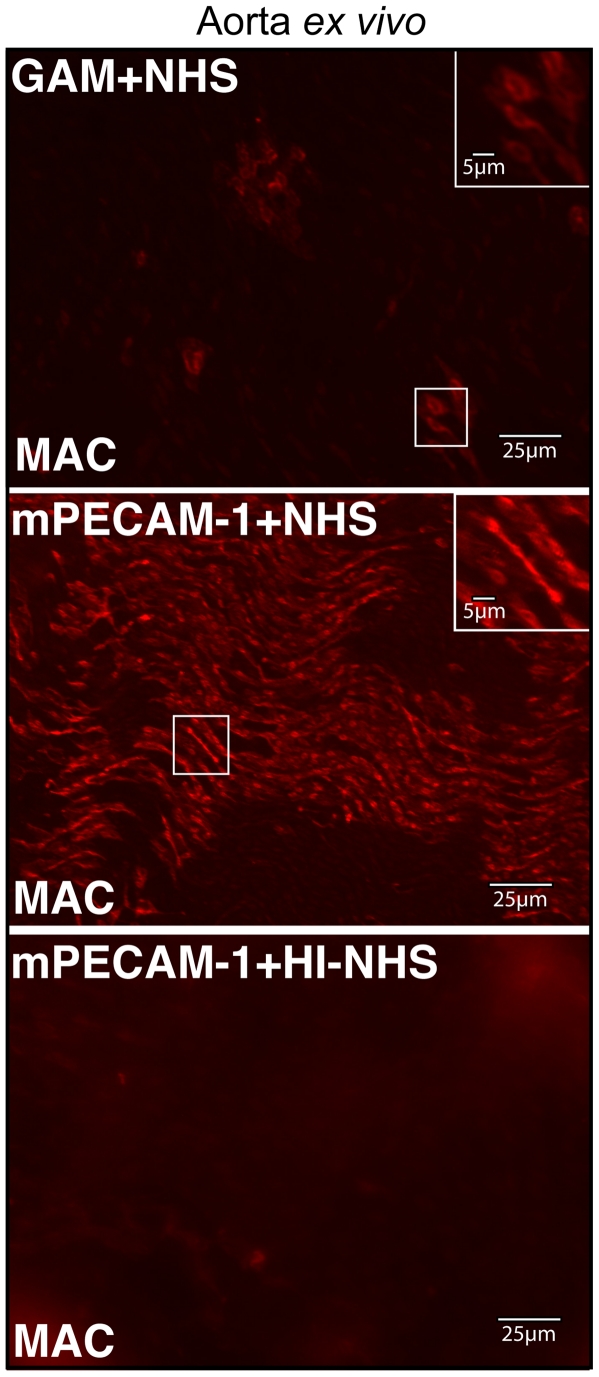
Cell-specific antibody is required for deposition of human MAC on endothelial cells of murine aorta *ex vivo*. Representative images are shown of flatmounts of murine aorta stained for human MAC following incubation with normal human serum and either a generic anti-mouse (GAM) antibody or mPECAM-1 antibody. As a control, staining of aorta for MAC was performed following incubation with mPECAM-1 antibody and heat-inactivated NHS (HI-NHS). While treatment with GAM and NHS results in only a few endothelial cells staining for MAC across the lumen of the aorta, incubation with mPECAM-1 antibody and NHS results in more extensive and uniform staining of endothelial cells across the tissue. Insets show higher magnification of boxed regions. Aorta incubated with mPECAM-1 antibody and HI-NHS shows little or no MAC staining.

To determine whether human complement is activated by murine endothelial cells, an explant of murine aorta was incubated with either an antibody against murine platelet/endothelial cell adhesion molecule (mPECAM-1) or a generic anti-mouse (GAM) antibody and subsequently incubated with either normal human serum (NHS) or heat inactivated NHS (HI-NHS). The tissue was subsequently stained for the presence of the membrane attack complex (MAC) deposited on cell surfaces, using an antibody against sC5b-9. In the presence of either NHS alone (data not shown) or GAM+NHS, low levels of MAC staining were observed on individual endothelial cells of the luminal surface (Figure 1). However, staining was limited to only a few patches of cells. Aorta that had been incubated with mPECAM-1+NHS exhibited significantly more robust and homogeneous deposition of MAC on endothelial cells across the luminal surface (Figure 1). Aortal tissue incubated with HI-NHS subsequent to incubation with mPECAM-1 antibody did not deposit MAC on its surface (Figure 1).

### Intracardial delivery of mPECAM-1 antibody results in binding of antibody to a variety of murine tissues *in vivo*

**Figure 2:**
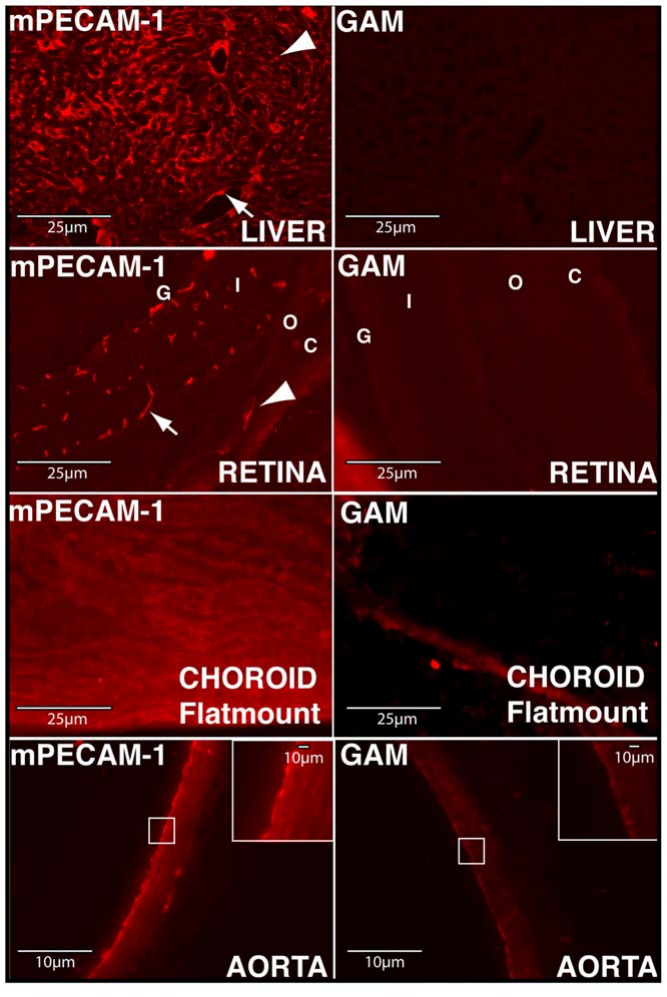
Intracardial delivery of mPECAM-1 antibody permits binding to a variety of murine tissues. Liver: In the liver, mPECAM-1 antibody binds endothelial cells along the sinusoids (arrowhead), as well as those of larger blood vessels (arrow). Retina: In the posterior eyecup, mPECAM-1 binds endothelia of the choriocapillaris (arrowhead) and retinal vasculature (arrow). Choroid: A flatmount of the choroid/RPE harvested from Balb/C mice injected intracardially with mPECAM-1 antibody shows more clearly binding of the antibody to the choroidal endothelium. Aorta: In the aorta, mPECAM-1 antibody is observed to bind the endothelial cell layer on the luminal surface (higher magnification of boxed region shown in inset). Intracardial delivery of a generic anti-mouse (GAM) antibody does not result in labeling of endothelial cells in any of the tissues. G, Ganglion Cell Layer; I/O, Inner/Outer Nuclear Layer; C, Choroid.

Due to the need for an endothelial cell surface antibody for activation of human complement by murine endothelial cells, we determined the feasibility of intracardial delivery of mPECAM-1 antibody into the systemic vasculature of mice. Four hours post- intracardial injection of either the mPECAM-1 antibody (or a GAM antibody), tissues were harvested and stained with a Cy3-conjugated goat anti-hamster antibody. mPECAM-1 staining was detected on a variety of tissues, including the liver, retina, choroid and aorta (Figure 2). In the liver, intracardial injection of mPECAM-1 antibody resulted in antibody binding to endothelial cells of both the sinusoids and larger blood vessels (arteries and veins). In the posterior eyecup, mPECAM-1 antibody was detected on endothelial cells of the retinal vasculature, as well as on those of the choriocapillaris of the choroid. To facilitate detection of antibody binding to endothelial cells of the choriocapillaris, which could be hampered by the presence of choroidal pigment, mPECAM-1 antibody was delivered intracardially to Balb/C mice and choroidal/RPE flatmounts prepared. With this procedure, extensive binding of mPECAM-1 antibody binding of the choriocapillaris could be observed. In the aorta, robust staining of endothelial cells of the luminal surface was observed. Intra-cardial delivery of GAM indicated no staining specific to the endothelium in the liver, retina, choroid, or aorta (Figure 2).

### Intracardial delivery of mPECAM-1 and NHS results in deposition of human MAC on endothelial cells of the liver

**Figure 3:**
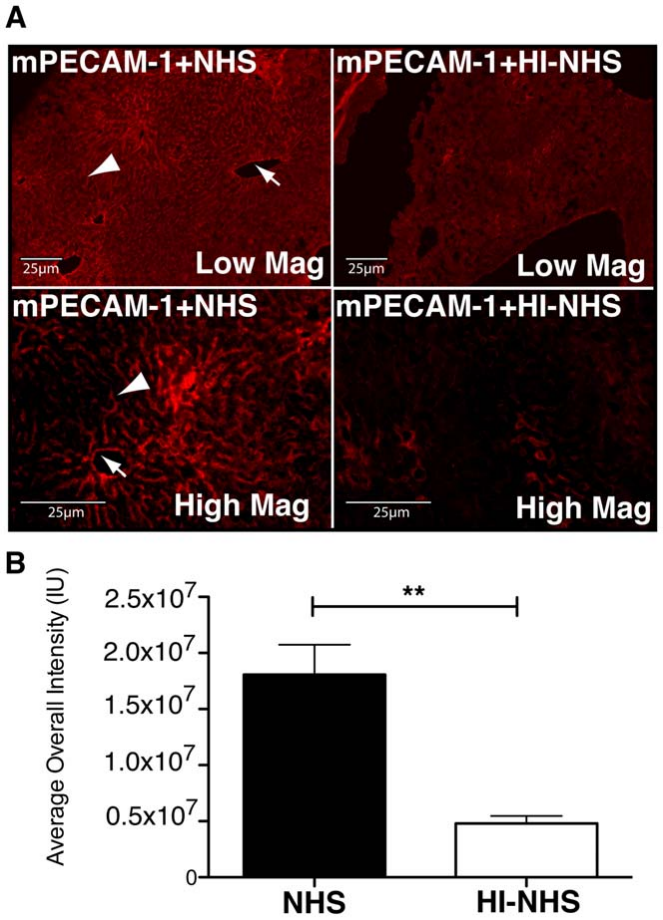
Intracardial delivery of mPECAM-1 and NHS results in deposition of human MAC on endothelial cells of blood vessels in murine liver. (A) Perfusion of liver with mPECAM-1 antibody and NHS results in MAC deposition on endothelial cells of sinusoid canals (arrowhead) and larger blood vessels (arrow). Liver perfused with mPECAM-1 and HI-NHS exhibits significantly less MAC deposition. (B) The average MAC staining intensity of livers of mice perfused with mPECAM-1 antibody and NHS is 3.8-fold higher (**p<0.01) than the staining intensity of those perfused with mPECAM-1 antibody and HI-NHS (n = 3).

Four hours post-intracardial delivery of mPECAM-1 antibody, mice were perfused with either NHS or HI-NHS. 15 minutes post serum infusion, tissues (liver, choroid, and retina) were harvested and stained for deposition of human MAC using an antibody against sC5b-9. Staining of liver sections from mice infused with mPECAM-1 and NHS exhibited prominent MAC deposition on the endothelial cells of sinusoids and larger blood vessels (Figure 3A). Liver sections of mice perfused with mPECAM-1 antibody and NHS (mPECAM-1+NHS) indicated significantly higher levels of MAC deposition relative to liver sections of control mice perfused with mPECAM-1 antibody and HI-NHS (mPECAM-1+HI-NHS, Figure 3B). After quantitation of MAC staining intensity, a 3.8-fold increase in MAC deposition was observed in the livers of mice injected with mPECAM-1+NHS (1.81×10^7^ IU) relative to the livers of mice injected with mPECAM-1+HI-NHS (0.48×10^7^ IU, p<0.01). Deposition of MAC was not detected in any of the other tissues examined (data not shown).

### Intraperitoneal injection of an adenovirus expressing human soluble CD59 protects against human MAC deposition on endothelial cells of murine liver vasculature

**Figure 4:**
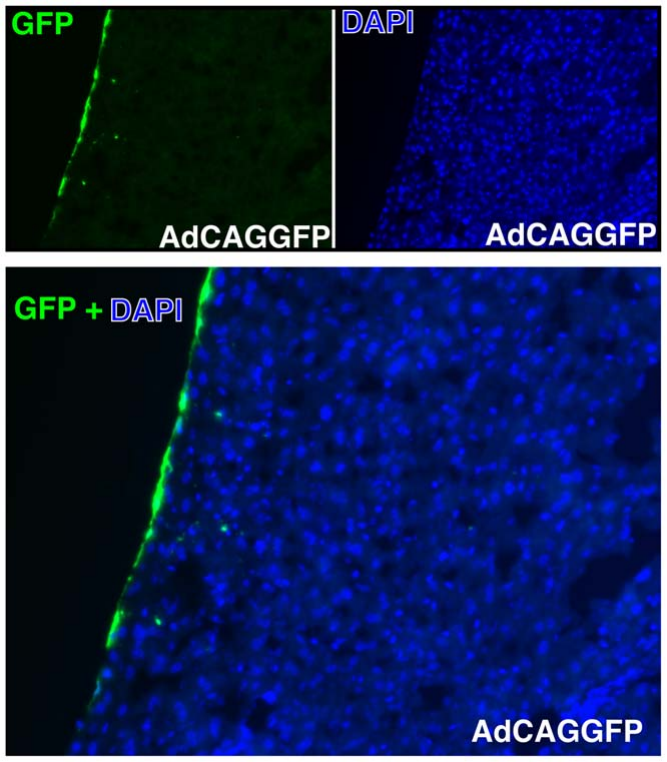
Intraperitoneal injection of an adenovirus expressing GFP (AdCAGGFP) shows significant transduction of murine liver at 7 days post-injection. A representative image of a transverse section of mouse liver showing GFP transduction is presented. DAPI stain of cell nuclei of the same liver section is also shown. GFP+DAPI overlay shows that GFP expression was observed almost exclusively along the peritoneal membrane, with a few cells within the liver expressing GFP.

**Figure 5:**
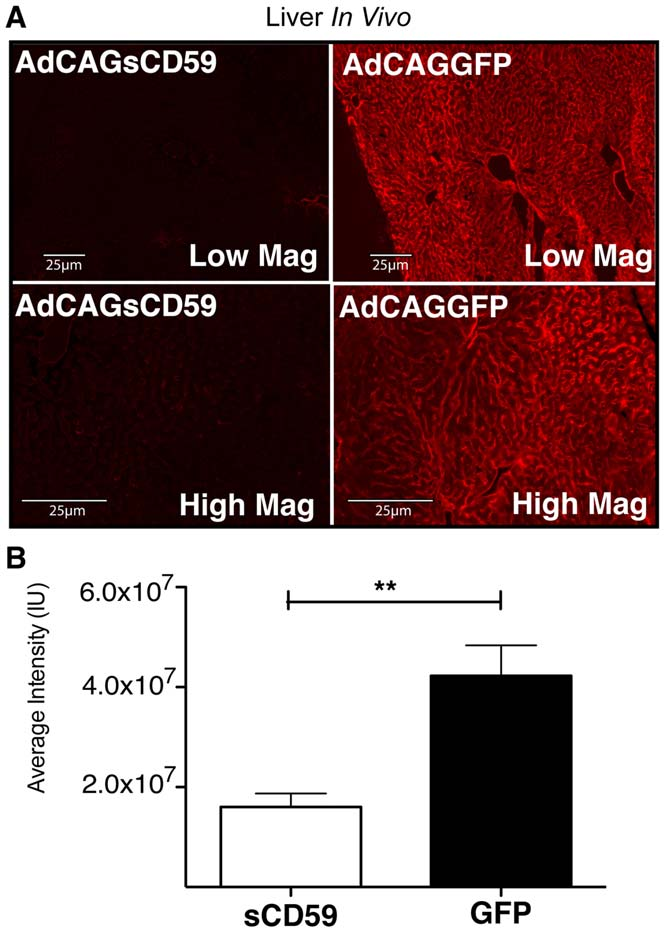
Intraperitoneal injection of an adenovirus expressing human sCD59 results in reduced deposition of human MAC on endothelial cells of liver vasculature. (A) Representative micrographs showing human MAC deposition on sinusoidal endothelial cells, as well as the endothelial cells of blood vessels in livers of mice perfused with mPECAM-1 antibody and NHS 7 days post-injection with an adenovirus expressing either sCD59 (AdCAGsCD59) or GFP (AdCAGGFP). Low and high magnification images are shown for each. The intensity and area of MAC staining is reduced in the AdCAGsCD59-treated mice. (B) The average MAC staining intensity of liver vasculature in AdCAGsCD59-injected mice is reduced by 62.1% (**p<0.01) relative to that of AdCAGGFP-injected mice (n = 8).

Intraperitoneal injection of an adenovirus expressing green fluorescent protein (GFP, AdCAGGFP) results in GFP expression mainly along the peritoneal membrane of the liver, with some expression of GFP by a few cells within the liver (Figure 4). A similar virus was generated expressing human soluble CD59 (sCD59), which lacks the C-terminal 26 amino acids encoding the signal sequence for attachment of the GPI anchor (AdCAGsCD59). Mice were administered either AdCAGsCD59 or AdCAGGFP by intraperitoneal injection and 7 days later injected intracardially with mPECAM-1 antibody followed by NHS as described above. 15 minutes after serum delivery, livers were harvested and sections stained for deposition of human MAC. While blood vessels and sinusoids of liver sections from mice treated with AdCAGGFP stained strongly positive for MAC (Figure 5A), blood vessels and sinusoids of liver sections from AdCAGsCD59-injected mice indicated little or no MAC staining (Figure 5A). Quantitation of staining indicated that the average MAC staining intensity was reduced by 62.1% (p<0.01) in AdCAGsCD59-injected mice relative to those injected with AdCAGGFP. The average MAC staining intensity of liver sections of the AdCAGsCD59-treated mice was 1.60×10^7^ IU relative to 4.23×10^7^ IU of those treated with AdCAGGFP (Figure 5B).

Because adenovirus has previously been shown to activate mouse complement [25] and the sC5b-9 antibody used can detect both mouse and human MAC, we performed a control experiment to exclude the possibility that any of the MAC deposited is from activated mouse complement. To address this, we injected mice intraperitoneally with AdCAGGFP 7 days prior to intracardial injection with mPECAM-1 antibody. Four hours later, instead of NHS, mice were administered PBS^++^ by intracardial perfusion. 15 minutes following PBS^++^ infusion, livers were harvested and stained for MAC using the sC5b-9 antibody. There was no MAC staining detected in any cell type in these livers (data not shown), confirming that the MAC deposition detected post-adenoviral injection in mPECAM-1+NHS treated mice is formed from human, not mouse, complement.

### AdCAGsCD59 protects against MAC deposition on endothelial cells of blood vessels of the liver

**Figure 6:**
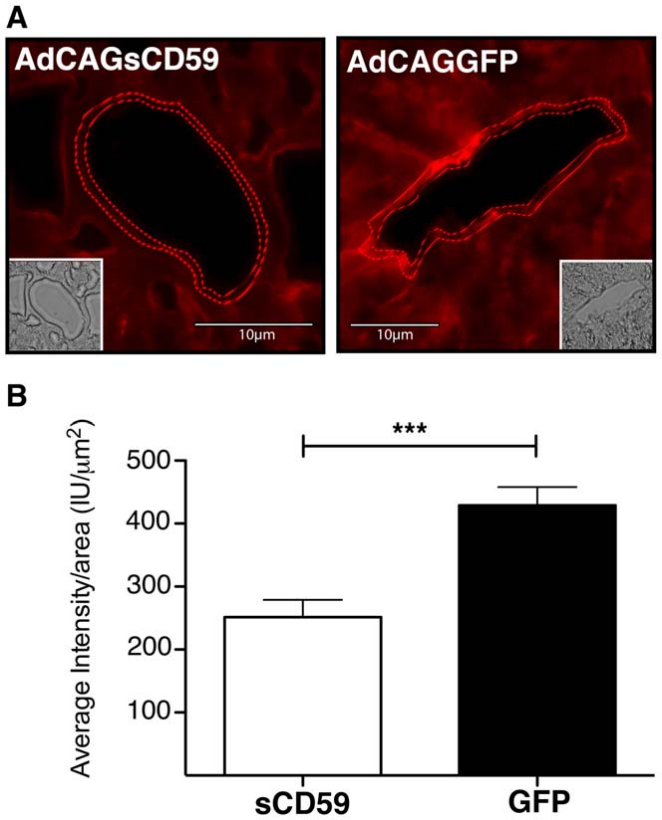
AdCAGsCD59 protects against human MAC deposition in the larger (non-capillary) blood vessels of the liver. (A) Representative images indicating human MAC staining on the endothelial cell layer of a large blood vessel of murine liver perfused with mPECAM-1 antibody and NHS 7 days post-injection with AdCAGsCD59 or AdCAGGFP. Corresponding brightfield images are also shown (inset). (B) The average MAC staining intensity per endothelium area of large liver vessels of AdCAGsCD59-injected mice is reduced by 41.4% (***p<0.001) relative to that of AdCAGGFP-injected mice (n = 8).

While MAC deposition was observed on endothelial cells of both the sinusoids and blood vessels, we were specifically interested in the protection conferred by sCD59 in endothelial cells of the blood vessels. Representative sections taken from livers of AdCAGsCD59- and AdCAGGFP-injected mice indicated that staining along the luminal surface of larger vessels in the AdCAGsCD59-treated mice was discontinuous and less intense relative to that of AdCAGGFP-injected mice (Figure 6A). Quantitation of staining on the hepatic blood vessels of AdCAGsCD59-injected mice indicated reduced MAC deposition (41.4%, p<0.001) relative to those of AdCAGGFP-treated mice. The average staining intensity per endothelial cell area in the livers of AdCAGsCD59-injected mice was 251.27 IU/µm^2^, while that of the vessels of livers of AdCAGGFP-injected mice was 428.95 IU/µm^2^.

## Discussion

The only FDA approved therapeutics targeting complement include an antibody against complement component 5 (eculizumab) and C1 inhibitor, but there are a number of ongoing trials investigating the safety and potency of complement regulators (reviewed in [26]). These include a peptide antagonist of complement component 3 (POT-4), an antibody against Factor D (TNX-234), and an aptamer targeting complement component 5 (ARC1905). Unlike the above listed complement regulators, CD59 specifically inhibits the terminal stage of complement activation, i.e. MAC formation on the plasma membrane. CD59 binds the C5b-8 protein complex deposited on the membrane and prevents the incorporation of multiple C9 molecules which are required for formation of the pore [27]. Specifically targeting MAC formation using CD59 has the advantage of keeping the upstream processes of the complement pathway intact for the purpose of fighting pathogens [28], [29], [30], as well as maintaining its important roles in tissue homeostasis [31].

We have shown previously that human GPI-anchored CD59 delivered via an adenovirus vector to murine RPE cells can provide robust protection to those cells against human MAC deposition [22]. However, protection was only conferred to those cells transduced by the adenovirus. In the plethora of disorders in which a major role of complement activation has been implicated, it is unlikely that a gene delivery vector will be available to transduce the variety of cell types subject to complement attack. We therefore elected to re-consider the capacity of a soluble version of CD59 as an anti-complement therapy when administered via a gene therapy approach. Soluble forms of CD59 have been described previously, but have been shown to be inefficient inhibitors of MAC both *in vitro* and *in vivo* [16], [19]. Those authors have speculated that sCD59's ineffectiveness *in vivo* may be due to its small size, which may make it more easily filtered by the renal system, as well as its inability to associate with the membrane, the site of MAC formation. We have recently shown, however, that sCD59 delivered by either an adenovirus or an AAV vector to murine ocular tissues can significantly reduce MAC deposition and laser-induced choroidal neovascularization in a model of neovascular AMD [21]. This result, unanticipated based on prior studies, suggested that sCD59 warrants further investigation as a treatment, not only for AMD and ocular disorders of complement, but also for disturbances of complement activity affecting other tissues.

In this study, we have confirmed the potential of sCD59 delivered using a gene therapy approach to protect other tissues, such as liver. In addition, we have demonstrated the ability of sCD59 to protect cells, in this case endothelial cells, remote from the site of vector transduction. Intraperitoneal injection of an adenovirus expressing human sCD59 resulted in viral transduction mainly along the peritoneal membrane at the periphery of the liver. As sCD59 is produced, we postulate that it diffuses through the extracellular matrix (ECM) towards the vasculature. We further speculate that this mode of delivery will cause an accumulation of sCD59 in the ECM around the endothelial cells, potentially enhancing the probability of contact with C5b-8. That protection is likely provided by sCD59 residing in the ECM or close to the endothelial cell surface, and not sCD59 in the circulation, is supported by the study design whereby we flush out the mouse blood prior to perfusion with human serum. A similar observation has been made for protection of brain capillary endothelium from complement activation by C1 inhibitor in a murine model of transient brain ischemia [32]. With this in mind, we consider that this treatment approach is most appropriate for those diseases in which complement activation occurs in endothelial and/or other cell types of a specific tissue, such as is the case in organ transplantation, AMD, diabetic retinopathy, aHUS, and dense deposit disease. In future studies we would like to determine whether or not sCD59 which is expressed from an adenovirus vector residing in the liver and which enters the bloodstream retains sufficient potency to prevent MAC deposition on endothelial cells of the systemic vasculature, a finding particularly relevant to atherosclerosis and systemic complement activation associated with AMD.

Viral vectors such as adenovirus have been demonstrated to express transgene products for the lifespan of mice [33] and have been observed to exhibit a broad of tropism for different tissues, particularly liver and kidney following intraperitoneal injection [34]. However, we envisage that this approach could be extended to AAV vectors which are generally considered safer, due to their lower immunogenicity, for use in humans.

Inappropriate activation of complement is an underlying cause of many diseases, and a number of these are known to involve complement-mediated damage of endothelial cells [1]. An *ex vivo* xenotransplantation simulation assay has previously shown human MAC deposition along porcine endothelial cells [10]. Herein, we have established an *in vivo* model of human MAC deposition on endothelial cells of murine liver vasculature. This model may be useful in evaluating anti-complement treatments for diseases in which complement-induced damage to endothelial cells is known to play a role. While caution needs to be exercised when data from animal studies is used as an impetus to proceed to the clinic, as many of the observations made in animal models have not been confirmed in humans, the model employed in this study has the advantage of permitting evaluation of anti-complement therapies against human complement activation in an *in vivo* setting.

Despite detection of the perfused “activating” PECAM antibody binding to endothelial cells in the other tissues examined (retina, choroid, and aorta), we were unable to detect deposition of MAC in any of these tissues. This may be due to differences in local concentrations of endogenous complement regulators, such as CD59, between these tissues. For example, murine CD59a has shown some protection against human complement *in vitro* [35]. Further development of this model could include reproducing this assay in mice deficient in one or more endogenous complement regulators, such as mice deficient in CD59a/b [36].

In summary, we have generated an *in vivo* model of human MAC deposition on endothelial cells of murine liver vasculature. This model should prove useful for the testing of anti-complement therapies for diseases known to involve complement-mediated damage to endothelial cells, such as aHUS, dense deposit disease, AMD, and diabetic retinopathy. In addition, we have shown the potency of a non membrane-targeting sCD59 as a protective agent against complement attack when delivered via a gene therapy approach.

## Methods

### Animal use and care

Animal use in this study was in accordance with the ARVO Statement for the Use of Animals in Ophthalmic and Vision Research. This study was approved by Tufts University Institutional Animal Care and Use Committee (IACUC) protocol B2009-03 and Tufts University Institutional Biosafety Committee registration 2008-BRIA27. C57BL6/J and Balb/C mice aged 6–10 weeks were purchased from Jackson Laboratories (Bar Harbor, ME) and maintained in 12-hour dark light cycles in accordance with federal, state, and local regulations. Mice were anesthetized by intraperitoneal injection of 0.1 ml/10 g of ketamine (10 mg/ml)/xylazine (1 mg/ml).

### *Ex vivo* MAC deposition assay

C57BL6/J mice were anesthetized as described above. The thoracic cage was opened, and blood flushed with 5 mL of sterile PBS using a 21 gauge needle via the left ventricle. The descending aorta was identified, extracted, and flattened. After a brief incubation in ice-cold sterile PBS, the aorta was incubated in 50 µg/ml Goat anti-Mouse IgG (GAM) (Jackson ImmunoResearch Labs, West Grove, PA), 50 µg/ml Hamster anti-mouse PECAM-1 (mPECAM-1) (clone 2H8, 1.4 mg/mL, prepared as described previously [37]), or PBS for 1 hour at 4°C. Subsequently, the tissue was washed twice with ice-cold PBS for 5 minutes and incubated with either 50% normal human serum (NHS) (Complement Technology, Tyler, Tx) or heat-inactivated (56°C for 1 hr) normal human serum (HI-NHS) in PBS^++^ (0.15 mM Ca^+^, 0.5 mM Mg^+^) for 8 minutes at 37°C. Aorta was rapidly washed, and fixed overnight with 10% buffered formalin.

### Intracardial delivery of mPECAM-1 antibody

C57BL6/J mice were anesthetized as described above. The apex of the heart was located by palpation, and 200 µl of mPECAM-1 (250 µg) was delivered to the left ventricle using a 29 gauge needle. After 4 hours, mice were re-administered xylazine/ketamine, the thoracic cage was opened, and blood flushed with 5 ml of sterile PBS using a 21 gauge needle via the left ventricle. The descending aorta, the median and left lobes of the liver, and both eyes were harvested and fixed overnight at 4°C in 10% buffered formalin. For choroidal flatmounts, the same procedure was employed in Balb/C mice.

### Adenovirus construction

AdCAGsCD59 was constructed as previously described [21]. Briefly, the soluble form of CD59 was derived by manipulation of the membrane bound human CD59 cDNA (IMAGE ID #2988140, ATCC, Manassas, VA). Specifically, the sequence encoding the C terminal 26 amino acids, representing the signal sequence for attachment of the GPI anchor at Asp 77, was removed by PCR amplification and cloned into pShCAG [38]. The resulting plasmid was recombined with pAdEasy-1 [39] in BJ5183 to generate pAdCAGsCD59. 15 µg of pAdCAGsCD59 was linearized with PacI, ethanol precipitated, and transfected into 911 cells [40]. AdCAGsCD59 was propagated and purified using a virus purification kit (Puresyn, Malvern, PA) at a concentration of 0.9×10^11^ particles/ml. AdCAGGFP was constructed as previously described [38].

### *In vivo* MAC perfusion assay

C57BL6/J mice were injected with 5×10^9^ particles of AdCAGGFP or AdCAGsCD59 in 200 µl sterile PBS into the intraperitoneal cavity. After 7 days, injected and uninjected mice were anesthetized as described above. 200 µl of mPECAM-1 antibody was administered intracardially using a 29 gauge needle. After 4 hours, mice were re-anesthetized, the thoracic cage opened and blood flushed with 1 ml of sterile PBS using a 21 gauge needle via the left ventricle. Immediately, the mouse was perfused with 1.5 ml of 90% NHS or HI-NHS solution in PBS^++^, and incubated for 15 minutes at 37°C. The median and left lobes of the liver and both eyes were harvested and fixed overnight at 4°C in 10% buffered formalin.

### Tissue preparation

Fixed liver lobes and eyes were dehydrated by incubation in 15% and 30% sucrose. Liver lobes were trimmed to fit cryo molds and eyes were dissected to remove the anterior segments. Tissue was cryo-preserved and sectioned at 8 µm. For choroidal flatmount, fixed Balb/C mouse eyes were briefly washed in PBS. Eyes were dissected and the anterior segments were removed. The optic nerve was detached from the retinal junction. Retina was carefully peeled away from the posterior eyecup. Staining was performed as described below, with the exception that 1% PBS-Triton-X (PBS-T) was used in all incubations and washes. After staining, a Von Graefe Knife (2×30 mm; Miltex, Germany) was used to initially separate the RPE/choroid layer from the sclera. Separation was completed using a fine forceps to peel away the RPE/choroid layer, sliding the knife underneath to prevent tearing of the tissue. The RPE/choroid complex was then flatmounted (Fluoromount-G; Southern Biotech, Birmingham, Al) and coverslipped.

### Immunofluorescent staining

Tissue slides were quickly dried and re-wetted with PBS. Slides were permeabilized and blocked with 6% goat serum (Jackson ImmunoResearch Labs) in 0.3% PBS-T for 30 minutes at room temperature. Samples and slides were subsequently incubated with Rabbit polyclonal anti-sC5b-9 neoantigen IgG (8.3 mg/ml, Complement Technology) at a dilution of 1∶400 in 0.3% PBS-T for 2.5 hours at room temperature. Slides were subsequently washed. Staining was completed by incubation with a CY3-conjugated Goat anti-Rabbit IgG (Jackson ImmunoResearch Labs) for 1 hour at room temperature. Slides were washed, flatmounted and coverslipped. MAC staining was also conducted using the same procedure with a separate antibody; mouse anti-human C5b-9 (clone aE11; Abcam, Cambridge, MA), was used at a 1∶100 dilution , while the secondary antibody, CY3 Goat anti-mouse IgG (Jackson ImmunoResearch Labs) was used at a 1∶400 dilution. Staining for mPECAM-1 antibody delivery was performed in a similar manner: slides were blocked with 6% goat serum in 0.3% PBS-T for 30 minutes at room temperature and subsequently incubated with a CY3 Goat anti-Armenian Hamster IgG (Jackson ImmunoResearch Labs), at a 1∶400 dilution in 0.3% PBS-T for 1 hr at room temperature. Slides were subsequently washed, flatmounted and coverslipped.

### Image analysis

Imaging was performed using control-matched settings on an Olympus IX51 microscope equipped with a Retiga 2000r camera. Images were processed identically with Photoshop CS2 (Adobe; Redwood City, CA). Overall Intensity and vessel intensity was measured using ImageJ (NIH; Bethesda, Md). All images to be analyzed were converted to 8-bit greyscale (intensity range = 0–255 IU). Background, as defined by max intensity in regions without tissue (i.e. between large blood vessels), was subtracted using the ‘Set Minimum’ function. To calculate overall intensity, images of regions with representative staining were analyzed using the ‘measure’ function to detect integrated density (total intensity). Two images were analyzed per lobe, resulting in four images averaged for each mouse (data point). Large blood vessels were defined as those with a diameter larger than two cell widths, and include arteries, arterioles, veins, and venules, but exclude capillaries and sinusoids. To calculate vessel intensity, freehand-selection tool was used to trace the outer boundary of the endothelial cell-layer cross section. A corresponding brightfield image was overlaid to ensure accurate region selection. Total intensity (I_outer_) and total area (A_outer_) was calculated using the measure function. The inner boundary of the endothelial cell-layer cross-selection was subsequently traced and the total intensity (I_inner_) and total area (A_inner_) measured. Area was converted to µm^2^ with the scale of 2.727 pixels/µm (200x zoom). To calculate the average intensity per area (X, in IU/µm^2^), the following equation was used: 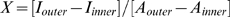. 10 random vessels were analyzed per lobe, resulting in 20 vessels averaged for each mouse (data point).
